# Loss of PI3 kinase association improves the sensitivity of secondary mutation of KIT to Imatinib

**DOI:** 10.1186/s13578-020-0377-9

**Published:** 2020-02-12

**Authors:** Guangrong Zhu, Jun Shi, Shaoting Zhang, Yue Guo, Ling Huang, Hui Zhao, Yideng Jiang, Jianmin Sun

**Affiliations:** 1 School of Basic Medical Sciences, Ningxia Medical University, No. 1160 Shengli Street, Yinchuan, 750004 China; 2 Key Laboratory for Regenerative Medicine, Ministry of Education, School of Biomedical Sciences, Faculty of Medicine, The Chinese University of Hong Kong, Hong Kong, Hong Kong SAR, China; 3 Kunming Institute of Zoology, Chinese Academy of Sciences-The Chinese University of Hong Kong Joint Laboratory of Bioresources and Molecular Research of Common Diseases, Hong Kong, Hong Kong SAR, China; 4 NHC Key Laboratory of Metabolic Cardiovascular Diseases Research (Ningxia Medical University), Yinchuan, China; 5 Ningxia Key Laboratory of Vascular Injury and Repair Research, Yinchuan, China; 6 Division of Translational Cancer Research, Lund Stem Cell Center, Department of Laboratory Medicine, Lund University, Lund, Sweden

**Keywords:** KIT, GISTs, PI3 kinase, Imatinib, Drug resistance

## Abstract

**Background:**

KIT mutations are the predominant driver mutations in gastrointestinal stromal tumors (GISTs), and targeted therapy against KIT has improved treatment outcome dramatically. However, gaining secondary mutation of KIT confers drug resistance of GISTs leading to treatment failure.

**Results:**

In this study, we found that secondary mutation of KIT dramatically increases the ligand-independent activation of the receptor and their resistance to the often used KIT inhibitor Imatinib in the treatment of GISTs. PI3 kinase plays essential roles in the cell transformation mediated by the primary mutation of KIT. We found that loss of PI3 kinase association, but not the inhibition of the lipid kinase activity of PI3 kinase, inhibits the ligand-independent activation of secondary mutations of KIT, and increases their sensitivity to Imatinib, and loss of PI3 kinase association inhibits secondary mutations of KIT mediated cell survival and proliferation in vitro. The in vivo assay further showed that the growth of tumors carrying secondary mutations of KIT is more sensitive to Imatinib when PI3 kinase association is blocked while inhibition of the lipid kinase activity of PI3 kinase cannot inhibit tumor growth, indicating that PI3 kinase is important for the drug resistance of secondary mutation of KIT independent of the lipid kinase activity of PI3 kinase.

**Conclusions:**

Our results suggested that PI3 kinase is necessary for the ligand-independent activation of secondary mutations of KIT, and loss of PI3 kinase association improves the sensitivity of secondary mutations to the targeted therapy independent of the lipid kinase activity of PI3 kinase.

## Background

Gastrointestinal stromal tumors (GISTs) are the most common mesenchymal tumors in the digestive tract, the incidence of GISTs is 4.3–21.1 cases per million per year depending on the population investigated. GISTs mainly arise in the stomach and small intestine, and to a lesser extent in large intestine, colorectum and esophagus, in addition, extragastrointestinal GIST was also reported but very rare [1]. Gene mutations in GISTs have been widely studied, the type III receptor tyrosine kinase KIT was identified as the most often mutated gene in GISTs, KIT mutations account for around 75% of GISTs [2–10]. In addition to KIT mutations, mutations of another type III receptor tyrosine kinase PDGFRA account for around 10% of GISTs [2, 3, 11]. Furthermore, mutations of SDH, HRAS, NRAS, BRAF and TF1 occur in GISTs as well but much less frequently [12].

Relapse of low risk primary GISTs after surgery is not very often, however, targeted therapy with Imatinib is necessary for the treatment of primary GISTs with high risk and metastatic GISTs. Imatinib is a small molecular inhibitor that can inhibit the activation of wild-type KIT and most KIT mutations identified in GISTs, and others such as PDGFRA and BCR-Abl. Treatment of GISTs with Imatinib can prolong the survival of GISTs patients for around 5 years in average [13], but most tumors will finally develop drug resistance by gaining a drug-resistant secondary mutation of KIT [14–19] or activation of alternative signaling pathways [20–23], leading to treatment failure. To overcome the drug resistance of GISTs to Imatinib, Sunitinib and Regorafenib have been developed and approved as the second line and third line targeted therapy of GISTs respectively. Compared with Imatinib which can prolong patient survival for years, the benefit from both Sunitinib and Regorafenib is very limited, they can prolong patient survival for only few months [24, 25].

Signal transduction of wild-type KIT has been widely studied. Wild-type KIT gets phosphorylated at certain tyrosine residues after ligand stimulation, phosphorylated tyrosines serve as docking sites for signaling molecules and activate downstream signaling pathways. Among the downstream signaling pathways, activation of Src family kinases and PI3 kinase is crucial for KIT signaling and KIT mediated biological response such as cell survival and proliferation [26]. Oncogenic mutations of KIT identified in malignancies such as mastocytosis and GISTs can be autoactivated without ligand stimulation, and the ligand-independent activation of KIT mutations is dependent on the PI3 kinase association with KIT, loss of PI3 kinase association dramatically inhibits the ligand-independent activation of KIT mutations [27, 28]. In addition to the ligand-independent activation, KIT mutations can activate different downstream signaling pathways compared with wild-type KIT. It is D816V, the often occurred KIT mutant in mastocytosis, but not the wild-type KIT, that can phosphorylate PIK3CD, SLAP, MITF and Xkr5, and phosphorylation of these molecules regulates KIT mutations mediated signal transduction and cell transformation in a way that is different from wild-type of KIT [27, 29–31], suggesting that KIT mutants have the activity that wild-type counterpart does not have.

To well understand the underlying mechanism as to how the secondary mutation of KIT mediated drug resistance in order for a better treatment of relapsed GISTs, we investigated the signal transduction of secondary mutation of KIT in this study. We found that loss of PI3 kinase association, but not PI3 kinase inhibitor, inhibits the ligand-independent activation of secondary mutation of KIT, and loss of PI3 kinase association improves the sensitivity of tumors carrying secondary mutation of KIT to Imatinib treatment. These results suggested a crucial role of PI3 kinase in secondary mutation of KIT mediated resistance to Imatinib, and a possible novel approach to overcome the drug resistance by blocking PI3 kinase association.

## Materials and methods

### Cytokines, antibodies and inhibitors

Recombinant human stem cell factor (SCF) was purchased from ORF genetics (Kópavogur, Iceland). Anti-KIT antibody KitC1 was purified as described before [32]. PE conjugated anti-KIT antibody was purchased from Biolegend (San Diego, CA). PI3 kinase subunit p85α antibody, phospho-Erk Thr202/Tyr204 antibody, Akt antibody, Erk antibody and HRP conjugated β-actin antibody were purchased from Santa Cruz Biotechnology (Dallas, TX). Phospho-Akt (Ser473) antibody was purchased from Cell Signaling Technology (Danvers, MA). Anti-pY antibody 4G10 and chemiluminescent HRP substrate were purchased from Millipore (Billerica, MA). HRP conjugated goat anti-mouse IgG antibody, HRP conjugated goat anti-rabbit antibody and HRP conjugated donkey anti-goat IgG were purchased from Bioss Antibodies (Beijing, China). KIT inhibitor Imatinib and PI3 kinase inhibitor Copanlisib were purchased from MedChemExpress (Monmouth Junction, NJ).

### Cell culture

EcoPack cells (Clontech) were grown in Dulbecco's Modified Eagle Medium supplemented with 10% fetal bovine serum, 100 units/ml penicillin and 100 μg/ml streptomycin. Ba/F3 cells (DSMZ) were grown in RPMI 1640 medium supplemented with 10% heat inactivated fetal bovine serum, 100 units/ml penicillin and 100 μg/ml streptomycin, and 10 ng/ml recombinant murine IL-3. In order to establish Ba/F3 cells stably expressing KIT, EcoPack cells were transfected with KIT in pMSCVpuro vector, supernatants were used to infect Ba/F3 cells followed by selection with 1.2 μg/ml puromycin for 2 weeks, the expression of KIT in Ba/F3 cells were examined by flow cytometry and western blotting.

### Cell stimulation, immunoprecipitation (IP) and western blotting

Cell stimulation, immunoprecipitation and Western blotting were performed as described before [27].

### Cell survival and proliferation assay

Cell survival and proliferation assay was performed as described before [27].

### Animal experiments

Animal experiments were approved by the Animal Ethics Committee of Ningxia Medical University. Female nude mice (4–6 weeks old) were purchased from and maintained in the animal center of Ningxia Medical University. 6 × 10^6^ Ba/F3 cells expressing KIT mutant in PBS containing 10% Matrixgel were subcutaneously injected into right flank of nude mice. Tumor size was measured every day. When the tumor size reaches 100 mm^3^ (W557K558del/V654A) or 300 mm^3^ (W557K558del/V654A/M724A, these tumors grow slower, mice were treated with KIT inhibitor Imatinib (50 mg/kg) daily and/or PI3 kinase inhibitor Copanlisib (6 mg/kg) every other day for 10 days (4 mice per group). After sacrifice, the tumor size and weight were measured and tumor volume was calculated by (LxW2/2), L stands for the long side, W stands for short side.

## Results

### Secondary mutation increases the ligand-independent activation of KIT

Mutations of KIT in primary GISTs usually occur in exon 9, 11 and 13 while secondary mutations of KIT are usually identified in exon 13, 14, 17 and 18 in relapsed GISTs after treatment with Imatinib. To find the most often occurred primary and secondary mutations of KIT as work model, we searched literatures that sequenced KIT mutations across exon 9, 11 and 13 in primary GISTs and exon 13, 14, 17 and 18 in relapsed GISTs. 7 studies examined 346 primary GISTs and identified primary KIT mutations in exon 9, 11, 13 or 17 in 257 tumors (74.3%) [3, 5–10]. The deletion of W557K558 (W557K558del) in exon 11 and duplication of A502Y503 (A502Y503dup) in exon 9 are the most often occurred primary mutations of KIT, they respectively account for 11.3% and 9.7% of primary KIT mutations in GISTs (Table 1) GISTs carrying KIT mutations usually respond well to Imatinib, however, some tumors gain drug-resistant secondary mutation of KIT leading to relapse and treatment failure. By searching the literatures, 89 secondary mutations of KIT was reported in 143 Imatinib resistant tumors (62.2%) [14–19], the most often occurred secondary mutations are V654A in exon 13 and N822K in exon 17, they respectively account for 38.2% and 15.7% of Imatinib-resistant GISTs carrying secondary KIT mutations (Table 2). Thus, the mutations, e.g. W557K558del, A502Y503dup, V654A and N822K were used further as work models in this study.

**Table 1:** 110 mutations in exon 9, 11, 13 or 17 of KIT were identified in 257 of 346 primary GISTs

Mutation frequency	Mutations
29	W557K558del
25	A502Y503dup
19	V559D
17	V560D
7	D579del, V560del
6	W557R
5	K642E
4	557_561del, V559A, V559G, L576P, 574_586dup
3	550_558del, 551_556del, 552_553del, 552_557del, W557C/558_560 del, 558_562del, 564_576del, 569_576del
2	551_555del, 553_556del, 554_556del, 555_573del, 556_560del, K558I/V559del, V559del, 559_561del, V560G
1	K550L/551_560del, 550_554del, 550_556del, 550_557del/I558L559ins, 550_557del/K558G, P551L/M552del, 551_554del, M552K/553_556del, 552_558del, 552_570del, 552_570del/571insI, Y553N, Y553L/554_558del, 553_558del, 553_559del, E554D/555_560del, E554K/555_560del, 554_555del, V555del/Q556E, 555_556del, 555_559del, 555_560del/561insT, 555_571del, Q556V/W557T/558_559del, Q556H/557_560del, Q556H/557_572del, 556_557del/K558N, 556_560del/561insH, 556_569dup, 556_572del, 556_572del/573insH, 556_574del, W557G, W557F/K558Q, W557S/558_573del, W557T/558_563del, 557_558del/559insCE, 557_559del/V560F, 557_560del, 557_561del/562insFP, 557_564del, 557_574del, 557_559del/560insF, K558Q/V559P, K558R/559_564del, K558R/559_565del, K558del/559insNP, 558_559del, 558_559del/V560I, 558_564del, V559I, 559_560del, 559_568del/569insD, 560_576del, V560D/L576F, 561_575dup, 562_573del, 563_572del, 563_574del/575insGGGTCC, N567S, 567_573del, 572_577dup, 572_580dup, 572_583dup, 572_584dup, 573_578dup, 573_589dup, 574_580del, 574_585dup, 574_587dup, 574_591/591insA, Q575dup/L576V, 575_586dup, 576_586dup, 577_589dup, 577_591dup, 579_582dup/F584N, 579_587dup, 579_591dup, N822K

**Table 2:** 18 Mutations in exon 13, 14 or 17 of KIT were identified in 89 of 143 relapsed GISTs after Imatinib treatment

Mutation frequency	Mutations
34	V654A
14	N822K
11	Y823D
8	T670I
5	D820Y
4	D820G
3	D816H, D816E
2	C809G
1	643insA, S709F, K786N/D816H, D820A, D820E, D820V, N822D, N822Y, A829P

**Fig. 1:**
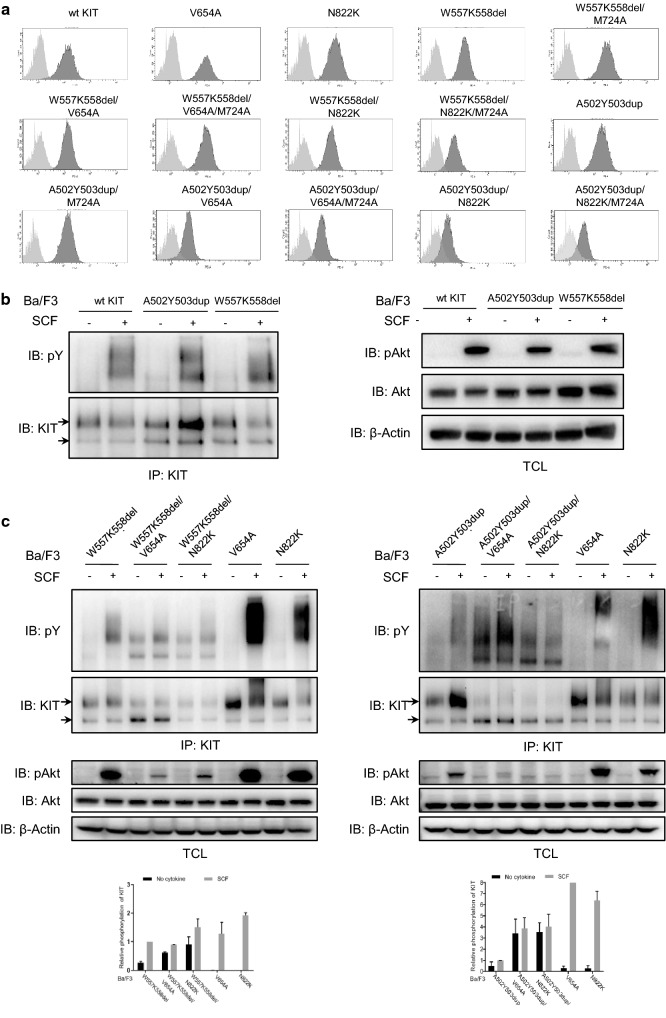
Secondary mutation increases the ligand-independent activation of KIT. **a** Wild-type KIT or KIT mutants in pMSCVpuro were transfected into EcoPack cells, supernatant was collected to infect Ba/F3 cells. After selection with puromycin, expression of KIT was examined by flow cytometry. Light gray: isotype control, dark gray: PE-anti-KIT antibody. **b** Ba/F3 cells stably expressing wild-type KIT or KIT mutants were washed and starved in RPMI 1640 medium for 4 h before stimulation with 100 ng/ml SCF for 2 min, KitC1 was used to precipitate KIT from cell lysate, after separation by SDS-PAGE and transfer to PVDF membrane, pY antibody 4G10 and KitC1 were used to detect KIT activation. pAkt, Akt and β-actin antibody were used to detect Akt activation in total cell lysates. **c** KIT activation was studied in Ba/F3 cells stably expressing primary and/or secondary KIT mutants as described above. Signal intensity was quantified and calculated to show the relative KIT activation

It has been reported that primary and secondary mutations of KIT usually occur in the same allele but not in two alleles respectively in relapsed GISTs after Imatinib treatment failure [14, 17, 33], which indicates that the KIT protein carries both primary mutation and secondary mutation in the relapsed tumor, we therefore established Ba/F3 cells stably expressing wild-type KIT and KIT mutants (Fig. 1a). After examination of KIT activation, we found that combination of primary mutation and secondary mutation dramatically increases the ligand-independent activation of KIT, while the ligand-independent activation of KIT with the single primary mutation or secondary mutation is much weaker and their full activation needs ligand stimulation (Fig. 1b, c). Since the ligand-independent activation of KIT mutations is considered as the cause of cell transformation, the increased ligand-independent activation of KIT carrying both primary mutation and secondary mutation might be the reason that primary mutation and secondary mutation occur in the same allele in relapsed GISTs in order for stronger activation of the receptor and increased malignancy of relapsed GISTs.

### PI3 kinase association is important for the ligand-independent activation of secondary mutation of KIT independent of the lipid kinase activity of PI3 kinase

**Fig. 2:**
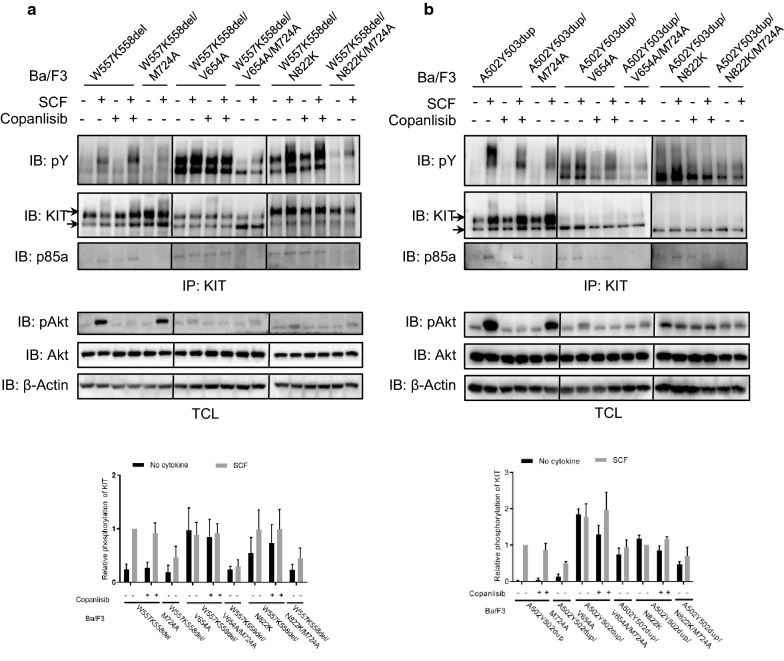
PI3 kinase association is necessary for the ligand-independent activation of secondary mutation of KIT. **a**, **b** Ba/F3 cells stably expressing KIT mutants were washed and starved in RPMI 1640 medium for 4 h in the presence of PI3 kinase inhibitor Copanlisib (50 nM). After stimulation with 100 ng/ml SCF for 2 min and cell lysis, KitC1 was used to precipitate KIT from cell lysate. pY antibody 4G10 and KitC1 were used to detect KIT activation. pAkt, Akt and β-actin antibody were used to detect Akt activation in total cell lysates. Signal intensity was quantified and calculated to show the relative KIT activation

PI3 kinases are a group of lipid kinases that play important roles in KIT mediated cell survival and proliferation [34–36]. In our previous studies, we found that loss of PI3 kinase association, but not inhibition of the lipid kinase activity of PI3 kinase, inhibits the ligand-independent activation of the often occurred KIT mutation V560D and D816V, and loss of PI3 kinase association inhibits both KIT/V560D and KIT/D816V mediated cell transformation [27, 28]. In addition, it has been reported that loss of PI3 kinase association blocks GISTs tumor development in knockin mice carrying germline KIT mutant [37]. In order to answer whether PI3 kinase is important for the activation of secondary mutation of KIT, we mutated the PI3 kinase binding site in KIT that block PI3 kinase binding. We found that, similar as primary KIT mutations, loss of PI3 kinase association dramatically inhibits the ligand-independent activation of secondary mutations of KIT (Fig. 2a, b). In contrary to that, PI3 kinase inhibitor did not affect KIT activation (Fig. 2a, b), suggesting that the inhibition of the ligand-independent activation of secondary mutations of KIT by loss of PI3 kinase association is independent of the lipid kinase activity of PI3 kinase, which is similar to the primary mutates of KIT [27, 28].

### Loss of PI3 kinase association increases the sensitivity of secondary mutations of KIT to Imatinib

**Fig. 3:**
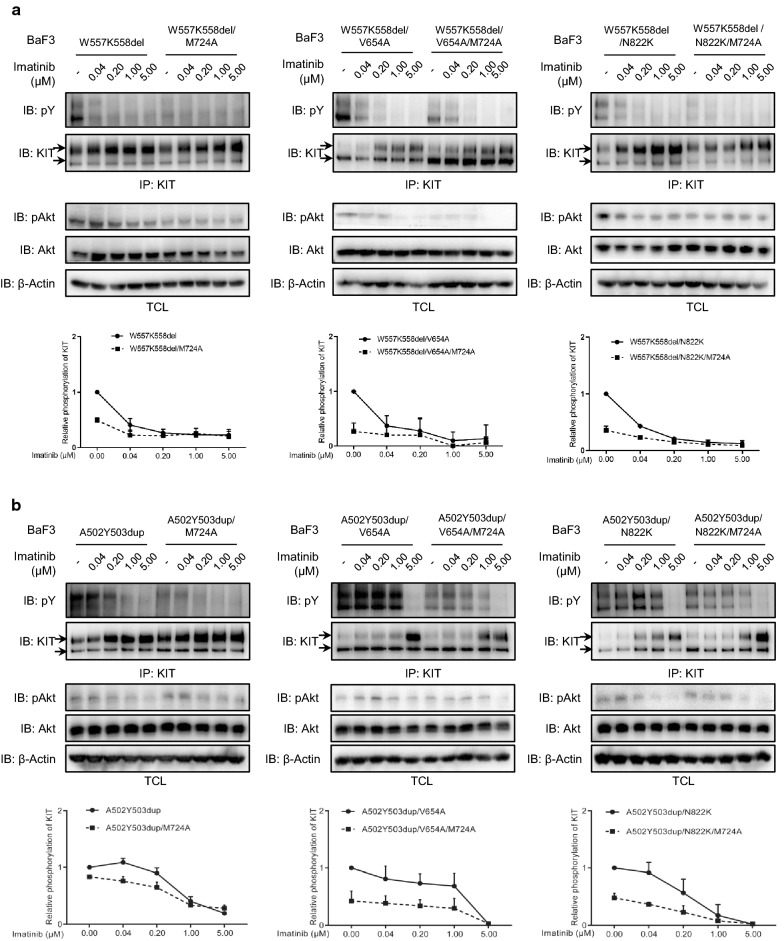

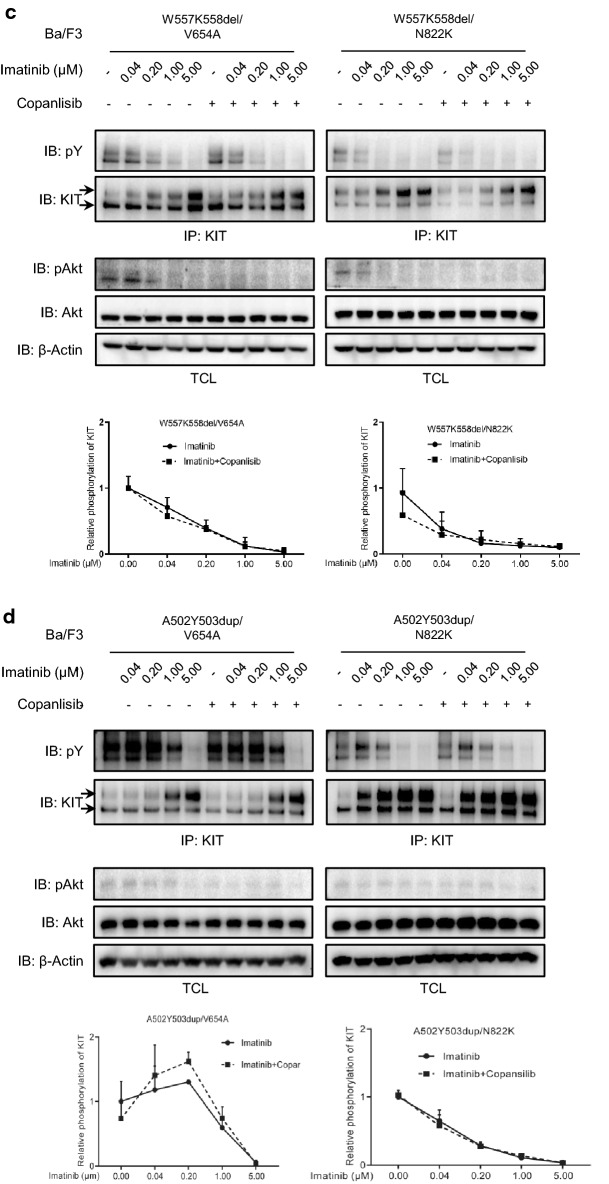
Loss of PI3 kinase association increases the sensitivity of secondary mutations of KIT to Imatinib. **a**, **b** Ba/F3 cells stably expressing KIT mutants with or without PI3 kinase association were washed, starved and incubated with various concentrations of Imatinib and **c**,** d** Copanlisib (50 nM) for 4 h. After stimulation with 100 ng/ml SCF for 2 min and cell lysis, KIT activation was detected by probe KIT immunoprecipitates with pY antibody 4G10 and KitC1. pAkt, Akt and β-actin antibody were used to detect Akt activation in total cell lysates. Signal intensity was quantified and calculated to show the relative KIT activation

GISTs can escape the inhibition of Imatinib by gaining the drug-resistant secondary mutation of KIT. Since loss of PI3 kinase association inhibits the ligand-independent activation of secondary mutation of KIT, we further examined the sensitivity of secondary mutation of KIT to Imatinib in the presence or absence of PI3 kinase association. As shown in Fig. 3a, b, the activation of secondary mutation of KIT were resistant to the inhibition of Imatinib. When PI3 kinase association was blocked, the activation of secondary mutation of KIT showed much lower kinetics, suggesting that loss of PI3 kinase association increases the sensitivity of secondary mutation of KIT to Imatinib. It has been reported that the sensitivity to Imatinib of exon 9 mutation A502Y503dup in primary GISTs is not as high as other often occurred primary KIT mutations, increased dose of Imatinib is recommended in the treatment of primary GISTs carrying KIT/A502Y503dup [38, 39]. In line with this report, we found that the secondary mutation of KIT in the presence of A502Y503dup is relatively insensitive to Imatinib (Fig. 3b), indicating that the sensitivity to Imatinib of primary mutation might have an impact on the sensitivity of secondary mutation to Imatinib.

Since PI3 kinase contributes to the ligand-independent activation of secondary mutation of KIT independent of the lipid kinase activity of PI3 kinase, we further examined that whether the important role of PI3 kinase in the sensitivity of secondary mutation of KIT to Imatinib requires the lipid kinase activity of PI3 kinase. As shown in Fig. 3c, d, PI3 kinase inhibitor does not change the sensitivity of secondary mutation of KIT to Imatinib, meaning that the lipid kinase activity of PI3 kinase is not required for the sensitivity of secondary mutation of KIT to Imatinib, which is similar as its important role in the ligand-independent activation of secondary mutation of KIT.

### Loss of PI3 kinase association inhibits secondary mutations of KIT mediated cell survival and proliferation

**Fig. 4:**
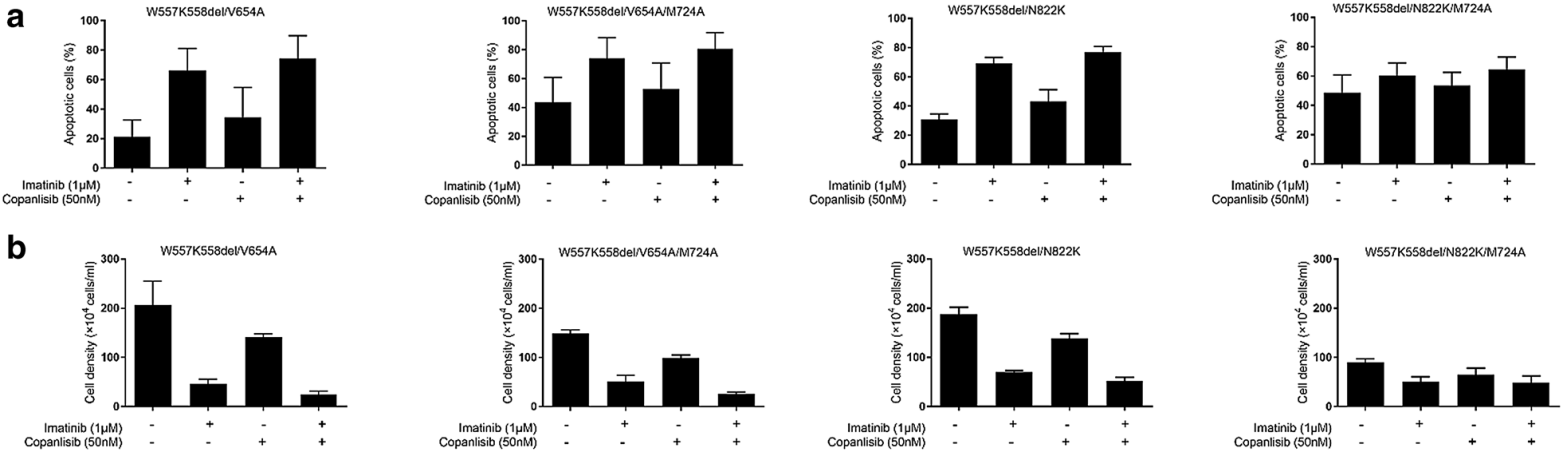
Loss of PI3 kinase association inhibits secondary mutations of KIT mediated cell survival and proliferation. Ba/F3 cells stably expressing KIT mutants with or without PI3 kinase association were washed and cultured in the absence of IL-3 for 48 h, **a** apoptotic cells were examined by flow cytometry after staining with Annexin V-PE and **b** living cells were counted under microscope

Since loss of PI3 kinase association can attenuate ligand-independent activation of secondary mutation of KIT and the resistance of the receptor to Imatinib, we next asked whether loss of PI3 kinase association can inhibit secondary mutation of KIT mediated cell survival and proliferation in the presence of Imatinib in vitro. By flow cytometry, we found that loss of PI3 kinase association strongly increases apoptosis of cells expressing secondary mutations of KIT (Fig. 4a), indicating the important role of PI3 kinase association in secondary mutations of KIT mediated cell survival. Interestingly, Imatinib treatment can further increase cell apoptosis although the activation of secondary mutations of KIT is resistant to the inhibition of Imatinib. Similar as cell survival, loss of PI3 kinase association dramatically inhibited secondary mutations of KIT mediated cell proliferation, and Imatinib can inhibit cell proliferation as well (Fig. 4b).

Unlike that loss of PI3 kinase association inhibits secondary mutation of KIT mediated cell survival, treatment cells expressing secondary mutation of KIT with PI3 kinase inhibitor didn’t inhibit cell survival (Fig. 4a), suggesting that the important role of PI3 kinase in secondary mutation of KIT mediated cell survival relies on the association of PI3 kinase with KIT but not the lipid kinase activity of PI3 kinase, which is in line with the results in KIT activation (Fig. 2). In the cell proliferation assay, PI3 kinase inhibitor inhibited the proliferation of cells expressing secondary mutation of KIT although not as much as Imatinib (Fig. 4b), these results suggested a key role of PI3 kinase association with KIT in the secondary mutation of KIT mediated cell survival and proliferation.

### Loss of PI3 kinase association inhibits tumor formation and improves the sensitivity of secondary mutations of KIT to Imatinib in vivo

**Fig. 5:**
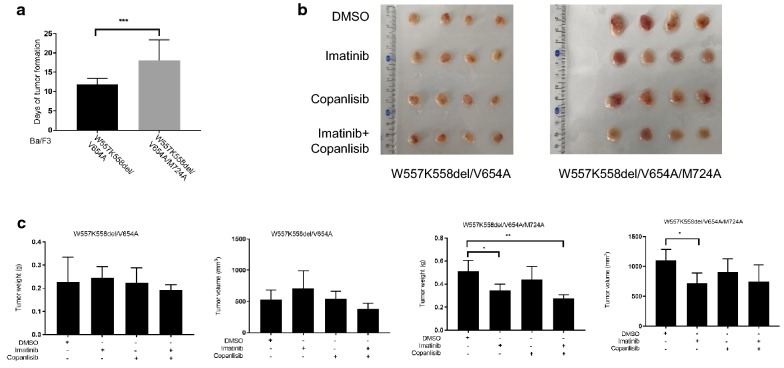
Loss of PI3 kinase association improves the sensitivity of secondary mutations of KIT to Imatinib in vivo. 6 × 10^6^ Ba/F3 cells stably expressing KIT mutant in PBS containing 10% Matrixgel were subcutaneously injected into right flank of nude mice. **a** The days of tumor formation were calculated. When the tumor size reaches 100 mm^3^ (W557K558del/V654A) or 300 mm^3^ (W557K558del/V654A/M724A), mice were treated with KIT inhibitor Imatinib (50 mg/kg) daily and/or PI3 kinase inhibitor Copanlisib (6 mg/kg) every other day for 10 days. After sacrifice, **b**, **c** the tumor size and weight were measured and tumor volume was calculated N = 4)

The resistance of secondary mutation of KIT to Imatinib is one of the reasons of GISTs treatment failure. In order to know whether PI3 kinase is important for the treatment of GISTs carrying secondary mutation of KIT in vivo, we established xenograft in nude mice. Tumors carrying secondary mutation of KIT were visible in around 11 days after injection while tumors carrying secondary mutation of KIT without PI3 kinase association were visible in 18 days after injection (Fig. 5a), indicating that loss of PI3 kinase association cannot block secondary mutation of KIT mediated tumor formation although it can delay the tumor formation. It has been reported that PI3 kinase association can block primary mutation of KIT mediated tumor formation completely [37], our results suggested a difference between primary and secondary mutation of KIT mediated tumor formation.

After a course of treatment of mice with Imatinib, we found that treatment of tumors carrying secondary mutation of KIT with Imatinib can’t inhibit the growth of tumor xenograft, while the tumor growth was inhibited by Imatinib when PI3 kinase association was lost (Fig. 5b, c), meaning that loss of PI3 kinase association improves the sensitivity of tumors carrying the secondary mutations of KIT to Imatinib treatment. It has been reported that PI3 kinase inhibitor alone can inhibit the tumor growth of primary GISTs and Imatinib can further increase the inhibition [40], our results showed that treatment of tumors carrying secondary mutation of KIT with PI3 kinase inhibitor alone or together with Imatinib does not further inhibit tumor growth although PI3 kinase inhibitor could inhibit cell proliferation in vitro (Fig. 4b), suggesting that the lipid kinase activity of PI3 kinase is not important for the resistance of secondary mutations of KIT to Imatinib, and the slight inhibition on the cell proliferation by PI3 kinase inhibitor in vitro is not enough to inhibit tumor growth in vivo. Furthermore, compared with Imatinib, treatment of tumors carrying secondary mutation of KIT with both Imatinib and PI3 kinase inhibitor did not give much more benefit regardless of PI3 kinase association with KIT, suggesting that patients with relapsed GISTs carrying secondary mutation of KIT probably cannot benefit from PI3 kinase inhibitor although in vitro study showed promising results (Fig. 4).

## Discussion

The treatment outcome of GISTs improved dramatically since the identification of KIT mutations in GISTs and application of small molecule inhibitor Imatinib in the treatment of GISTs in clinic. Following the success of Imatinib, numerous KIT inhibitors have been developed. So far, Imatinib, Sunitinib and Rogerafenib have been approved as the first, second and third line treatment of GISTs. Although GISTs patients can rely on Sunitinib and Rogerafenib after Imatinib treatment failure, Sunitinib and Rogerafenib can respectively extend the patient survival for only few months [24, 25], showing very limited benefit compared with that patients can survive for years when treated with Imatinib [13]. New treatment methods are still in urgent need for the treatment of relapse GISTs after failure of Imatinib.

KIT mutations in primary GISTs were mainly found in exon 9, 11 and 13 [3, 5–10] while exon 13, 14 and 17 are hotspots of KIT mutations in relapsed GISTs after treatment with Imatinib [14–19]. In this study, we showed that combination of primary mutation and secondary mutation of KIT can dramatically increase the ligand-independent activation of KIT. It is possible that the coexistence of two types of KIT mutation can further increase the ligand-independent activation of the receptor since it combines two different mechanisms to activate the receptor. In addition, although secondary mutation of KIT is the cause of drug resistance, we found that only secondary mutation of KIT without primary mutation cannot induce strong ligand-independent activation, meaning that secondary mutation itself may not be enough to induce drug resistance. That could be the reason why primary mutations and secondary mutations of KIT usually occur in the same allele.

PI3 kinase activation is often dysregulated in various types of cancers [41]. Among all subtypes, type IA PI3 kinases were most widely studied in cancer [42, 43]. Type IA PI3 kinases have two subunits, the regulatory subunit and the catalytic subunit. The regulatory subunit p85α or p85β usually bind to the catalytic subunit p110α, p110β or p110δ to form a heterodimer. However, there are more than 30% of p85 than p110 in the cells [44, 45], the free p85 can act as an adaptor and bind to other signaling molecules to regulate the signal transduction. Pan-PI3 kinase inhibitors and subtype-specific PI3 kinase inhibitors were developed and some of them have been approved for the treatment of cancers in clinic [41]. All these inhibitors can inhibit the lipid kinase activity of PI3 kinase and therefore inhibit the activation of PI3 kinase downstream signaling pathways. It has been reported that PI3 kinase is crucial for KIT/D816V induced cell transformation [46]. Unlike that the important role of PI3 kinase relies on its lipid kinase activity in cancers, we previously found that the key role of PI3 kinase in KIT mutation mediated cell transformation is independent on the lipid kinase activity of PI3 kinase [27, 28]. In this study, we further showed that the ligand-independent activation of secondary mutation of KIT relies on its association with PI3 kinase as well, and the oncogenic role of PI3 kinase in the secondary mutation of KIT mediated tumorgenesis is not dependent on the lipid kinase activity of PI3 kinase, loss of PI3 kinase increases the sensitivity of secondary mutation of KIT to Imatinib. These results suggest a possible novel approach to improve the treatment of KIT mutant induced malignancies by blocking the association of PI3 kinase with KIT but not application of PI3 kinase inhibitors. Furthermore, we found that loss of PI3 kinase association can further increase the sensitivity of primary KIT mutations to Imatinib although these mutations are not resistant to Imatinib, meaning that blocking the association of KIT and PI3 kinase might further improve the treatment outcome of primary GISTs as well.

Oncogenic mutations of receptor tyrosine kinases such as KIT, Flt3 and ALK can induce the ligand-independent activation of the receptor, and which is considered as the cause of cell transformation. In addition to the ligand-independent activation, it has been showed that oncogenic mutations of receptor tyrosine kinases can not only induce ligand-independent activation of the receptor, but also the signaling pathways of the oncogenic mutants are different from that of the wild-type counterpart [47]. For example, the internal tandem duplication of Flt3 (Flt3/ITD) can activate STAT5 which cannot be activated by wild-type Flt3 [48], KIT/D816V but not wild-type KIT can phosphorylate PIK3CD, SLAP, MITF and Xkr5 and thereby regulate KIT/D816V signaling and cell transformation in a way that is different from the wild-type of KIT [27, 29–31]. Similar as that, our current study suggests a key role of the non-lipid kinase activity of PI3 kinase in the activation of secondary mutations of KIT, and loss of PI3 kinase association with KIT but not PI3 kinase inhibitor can improve the sensitivity of secondary mutations of KIT to Imatinib treatment. Together with our previous studies that delineate the action of PI3 kinase in the signal transduction of primary KIT mutations, these data further suggest the difference between wild-type KIT and KIT mutants. The difference in the signaling between oncogenic mutations of receptor tyrosine kinases and their wild-type counterparts allow us to develop new therapeutic approaches by studying signaling pathways mediated by the oncogenic mutants but not by the wild-type receptor tyrosine kinases.

## Conclusions

Our results suggested that PI3 kinase is necessary for the ligand-independent activation of secondary mutations of KIT, and loss of PI3 kinase association improves the sensitivity of secondary mutations to the targeted therapy independent of the lipid kinase activity of PI3 kinase.

## Data Availability

Not applicable.
